# Supplementary material for: Soluble Urokinase-Type Plasminogen Activator Receptor (suPAR), Growth Differentiation Factor-15 (GDF-15), and Soluble C5b-9 (sC5b-9) Levels Are Significantly Associated with Endothelial Injury Indices in CAR-T Cell Recipients
Source: Int J Mol Sci. 2024 Oct 14;25(20):11028. doi: 10.3390/ijms252011028 (PMC11507105; doi:10.3390/ijms252011028)
Supplement: Supplementary file 1 [file ijms-25-11028-s001.zip › ijms-3201933-supplementary.pdf]

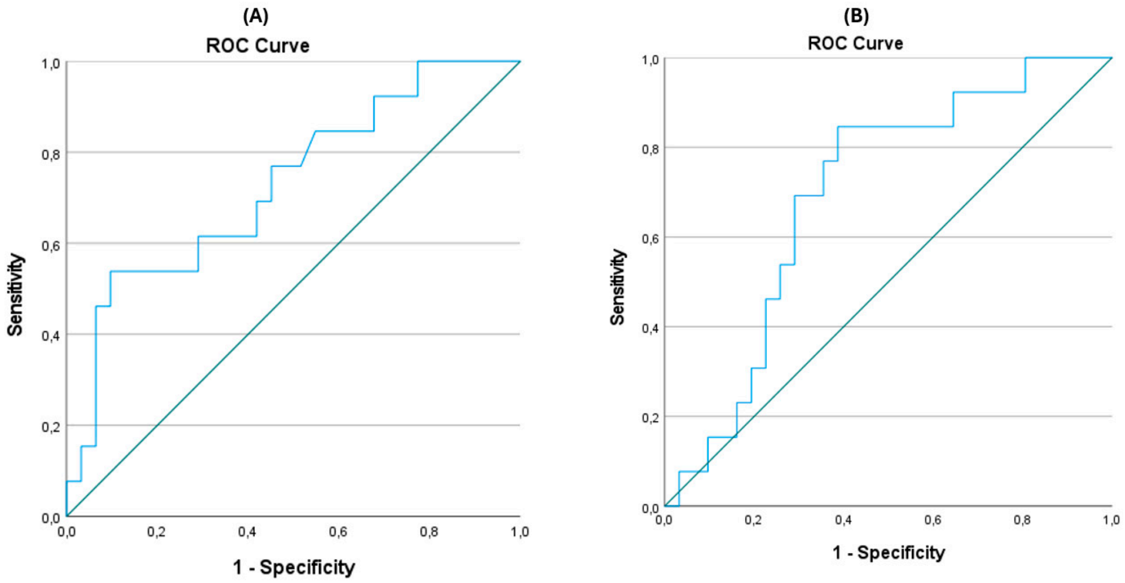

Figure S1. (A) S-EASIX0 (AUC: 0.728, p= 0.018), calculated at the day of the CAR-T cell product infusion, **(B)** and s-EASIX14 (AUC: 0.695, p=0.043), calculated 14 days post the infusion scores predicted the risk of death in ROC curves. s-EASIX= simplified Endothelial Activation and Stress Index, AUC= area under the curve, CAR-T= Chimeric antigen receptor-T, ROC= Receiver operating characteristic.

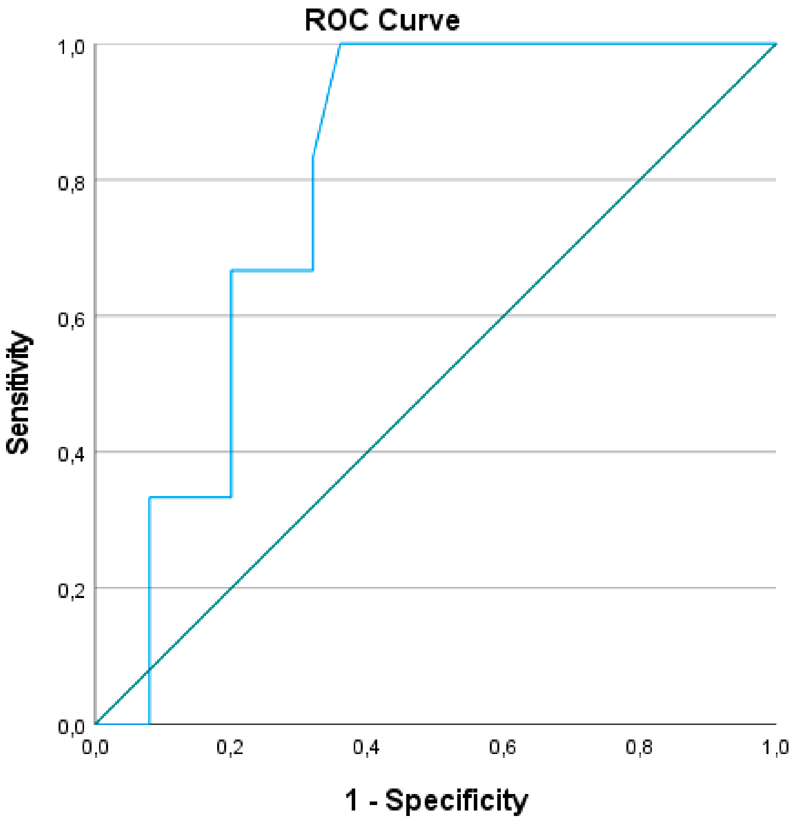

Figure S2. SC5b-9 levels at baseline predicted the risk of death 12 months post-infusion in the ROC curve (AUC: 0.797, p=0.026). sC5b-9= soluble C5b-9, AUC= area under the curve, ROC= Receiver operating characteristic.
